# Senescence-associated secretory phenotype and its impact on oral immune homeostasis

**DOI:** 10.3389/fimmu.2022.1019313

**Published:** 2022-10-04

**Authors:** Ziqi Yue, Lulingxiao Nie, Pengfei Zhao, Ning Ji, Ga Liao, Qi Wang

**Affiliations:** ^1^ State Key Laboratory of Oral Diseases, National Clinical Research Center for Oral Diseases, West China Hospital of Stomatology, Sichuan University, Chengdu, China; ^2^ Department of Prosthodontics, West China Hospital of Stomatology, Sichuan University, Chengdu, China; ^3^ Faculty of Dentistry, The University of Hong Kong, Sai Ying Pun, Hong Kong SAR, China; ^4^ Department of Information Management, Department of Stomatology Informatics, West China Hospital of Stomatology, Sichuan University, Chengdu, China

**Keywords:** oral homeostasis, senescence-associated secretory phenotype, cellular senescence, age-related disease, oral-systemic disease

## Abstract

The senescence-associated secretory phenotype (SASP), which accumulates over the course of normal aging and in age-related diseases, is a crucial driver of chronic inflammation and aging phenotypes. It is also responsible for the pathogenesis of multiple oral diseases. However, the pathogenic mechanism underlying SASP has not yet been fully elucidated. Here, relevant articles on SASP published over the last five years (2017–2022) were retrieved and used for bibliometric analysis, for the first time, to examine SASP composition. More than half of the relevant articles focus on various cytokines (27.5%), growth factors (20.9%), and proteases (20.9%). In addition, lipid metabolites (13.1%) and extracellular vesicles (6.5%) have received increasing attention over the past five years, and have been recognized as novel SASP categories. Based on this, we summarize the evidences demonstrating that SASP plays a pleiotropic role in oral immunity and propose a four-step hypothetical framework for the progression of SASP-related oral pathology—1) oral SASP development, 2) SASP-related oral pathological alterations, 3) pathological changes leading to oral immune homeostasis disruption, and 4) SASP-mediated immune dysregulation escalating oral disease. By targeting specific SASP factors, potential therapies can be developed to treat oral and age-related diseases.

## 1 Introduction

Oral immune homeostasis is a delicate balance established and shaped by the interaction between pathogen invasion and host immune response (1). Any disruption to this balance results in local or systemic diseases. Some pathophysiological changes are attributed to the environmental impact of senescent cells (2). The primary non-spontaneous effects of senescent cells appear to be closely linked to the senescence-associated secretory phenotype (SASP).

SASP, a product of senescent cells, is mainly classified into the following categories: 1) pro-inflammatory cytokines (such as interleukin (IL)-1α, IL-1β, IL-6, and IL-8); 2) chemokines (such as CXCL-1/3 and CXCL-10); 3) proteases: including matrix remodeling enzymes and plasminogen activators; 4) growth factors (such as VEGF, TGF-β and GM-CSF); 5) bioactive lipids (like oxidized lipid mediators); 6) extracellular vesicles (EVs); and 7) others (2, 3). SASP profiles exhibit a significant cell type-dependent heterogeneity, and SASP strength and composition are spatially and temporally dependent (4). In this paper, we summarize the different SASP categories, and reveal the most relevant cell types *via* bibliometric analysis, and propose a framework for the role of SASP in oral immune homeostasis to provide insights into the potential of SASP as a novel therapeutic target.

## 2 Methods

### 2.1 Data source and retrieval strategy

We searched the Web of Science Core Collection and PubMed databases for articles related to SASP factors published from 2017 to 2022. The retrieval strategy for the Web of Science Core Collection database was as follows: (((ALL=(cytokine) OR ALL=(chemokine) OR ALL=(protease) OR ALL=(growth factor) OR ALL=(lipid) OR ALL=(proinflammatory factor)) AND (ALL=(senescence associated secretory phenotype) OR ALL=(sasp))) AND (DOP==(2017-01-01:2022-04-01))) AND ((LA==(“ENGLISH”)) NOT (DT==(“REVIEW”))). The retrieval strategy for the PubMed database was as follows: (((((((cytokine) OR (chemokine)) OR (protease)) OR (growth factor)) OR (proinflammatory factor)) OR (lipid) AND ((y_5[Filter]) AND (English[Filter]))) AND ((senescence associated secretory phenotype) OR (sasp) AND ((y_5[Filter]) AND (English[Filter])))) NOT review[PT].

Inclusion criteria were as follows: Research articles 1. related to SASP; 2. published between 2017-01-01 and 2022-04-01; and 3. written in English. Exclusion criteria were as follows: 1. Literature whose content is not closely related to SASP factors; 2. Studies including guidance, consensus, industry standards, interviews, comments, announcements, advertisements, or letters to the editor; and 3. informally published studies, such as graduate theses.

### 2.2 Data processing and analysis

After retrieval, data screening and quality control were performed. This was conducted by reading titles and abstracts to remove literature that met the exclusion criteria. The data from papers that met the inclusion criteria were downloaded and merged. After removing duplicates, CiteSpace 6.1.R1 was used for data analysis. Next, different words or phrases expressing the same meaning were merged. For example, nuclear factor-kappa b and nf kappa b were merged as NF-kappa B. To clearly demonstrate the relationships among different type of SASP factors clearly, we further merged the same type SASP factors. For example, NF-kappa B, CCN1, and cyclin d1 were merged as proinflammatory factors, and stem cells, mesenchymal stem cells, and cancer stem cells were merged as pluripotent stem cells. Finally, a keyword co-occurrence network was built to visualize the relationships among knowledge domains and identify important SASP factors that have attracted attention in recent years.

## 3 Results

### 3.1 Analysis of the proportion of reported SASP factors

**Figure 1:**
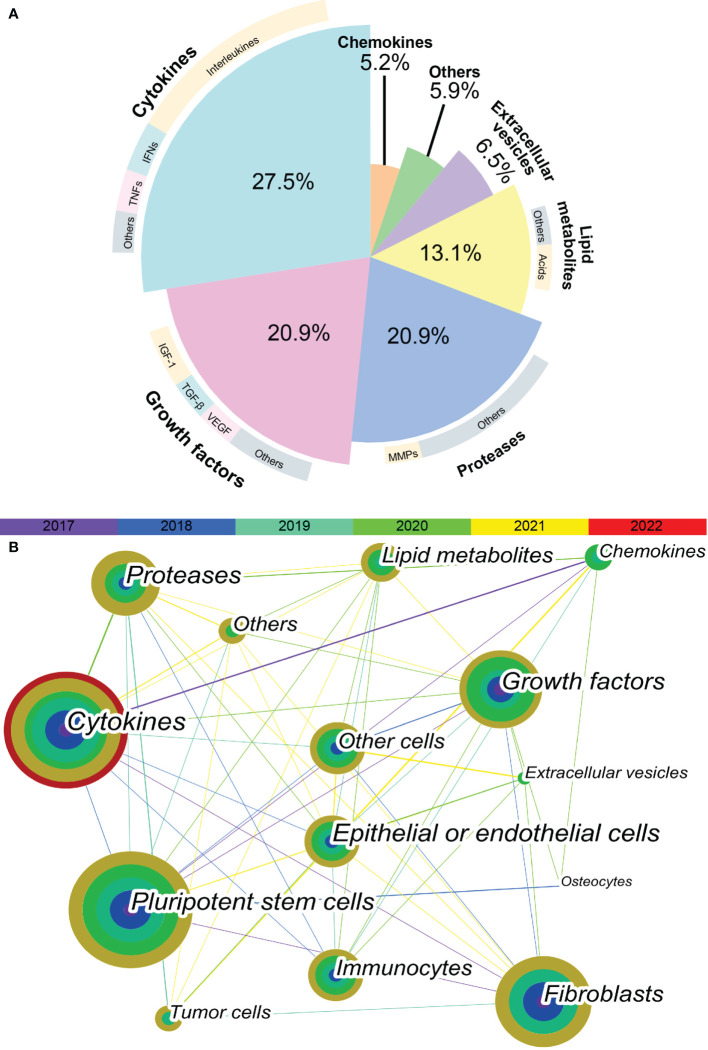
Keyword co-occurrence network visualization. **(A)** The proportion of different SASP factors in articles published in recent 5 years. Demonstrated as a pie plot. The inner are different types of SASP factors. The outer are most cited SASP factors of each type. **(B)** The keyword co-occurrence network shows the relationship between different types of SASP factors and different types of cells. Each node is a concentric circle, and the thickness of each layer of the concentric circle represents the frequency of use of this keyword in a certain year. The color of each line represents the year of the first co-occurrence between the two keywords. The thickness of each line represents the frequency of co-occurrence between the two keywords.

In total, 564 articles were included in the bibliometric analysis. In the last 5 years, the most cited SASP factors have been cytokines, including IL-6, IL-1, IL-8, CXCL-8; tumor necrosis factors (TNFs); and interferons. These proinflammatory cytokines account for 27.5% of the reported SASP factors. Among them, interleukins were determined to be the most important SASP factors, accounting for nearly half of these proinflammatory cytokines. The second-most cited SASP factors are growth factors and proteases. These two types of SASP factors account for 20.9% of variations in the SASP factors. IGF-1, TGF-β, and VEGF are the most frequently cited growth factors. Matrix metalloproteinases (MMPs) are the most cited proteases. In the past five years, more than half of the articles related to SASP have focused on various cytokines, growth factors, and proteases (**Figure 1A**).

### 3.2 Analysis of relevant cell type

As shown in **Figure 1B**, a tight and complex network of interactions is formed among different types of SASP factors and between SASP factors and different types of cells. The main cell types of interest in recent studies of SASP were pluripotent stem cells, fibroblasts, epithelial or endothelial cells, and immunocytes. The cell types in which the different SASP factors mainly act are listed in **Table 1**. The results of bibliometric analysis demonstrated that different SASP factors tend to affect different cell types. For example, cytokines mainly act on pluripotent stem cells, whereas proteases mainly act on tumor cells. Pluripotent stem cells, fibroblasts, and epithelial or endothelial cells are often affected by SASP factors.

**Table 1:** Varies cell types where different SASP factors mainly act on.

SASP factors	Cell types	Rank
Cytokines	Pluripotent stem cells	1^st^
	Immunocytes	2^nd^
	Fibroblasts	3^rd^
Growth factors	Pluripotent stem cells	1^st^
	Fibroblast	2^nd^
	Other cells	3^rd^
Proteases	Tumor cells	1^st^
	Pluripotent stem cells	2^nd^
	Immunocytes	3^rd^
Lipid metabolites	Epithelial or endothelial cells	1^st^
	Other cells	2^nd^
	Immunocytes	3^rd^
Extracellular vesicles	Other cells	1^st^
	Epithelial or endothelial cells	2^nd^
	Fibroblasts	3^rd^
Chemokines	Tumor cells	1^st^
	Osteocytes	2^nd^
	Pluripotent stem cells	3^rd^
Others	Pluripotent stem cells	1^st^
	Tumor cells	2^nd^
	Fibroblasts	3^rd^

This table shows the top 3 cell types that are most strongly affected by each type of SASP.

## 4 Discussion

### 4.1 Main components of SASP

#### 4.1.1 Cytokines

The most prominent cytokines are members of the IL-1, IL-6, and TNF families. The membrane-binding IL-1α is an upstream regulator of age-related cytokine networks (5). The secreted IL-1β is excreted from cells in the early stages of the inflammatory process and then binds to the IL-1 receptor to trigger an inflammatory response (6). IL-6 initiates intracellular signaling by binding to its membrane-binding receptor, IL-6Rα, or its soluble receptor, sIL-6R (7). An enhanced TNF signaling is considered pertinent to immune system defects (8).

#### 4.1.2 Chemokines

Chemokines act as local sensors of infection and inflammation (9). The most-studied chemokines in the field of aging in the past 5 years are the CXCL family members IL-8, CXCL-1, -2, and -3 and CCL family members like MCP-1, -2, and -4 and MIP-3α and-1α.

#### 4.1.3 Growth factors

The diffusion of growth factors into the surrounding environment induces cell activation and proliferation, stimulates granulation tissue formation, regulates inflammatory responses, induces angiogenesis, and participates in matrix remodeling and re-epithelialization (10).

#### 4.1.4 Extracellular proteases

*Matrix metalloproteinases (MMPs) and tissue inhibitors of metalloproteinases (TIMPs)* The MMP family is capable of degrading various components of extracellular matrix (ECM) proteins. TIMPs abrogate the proteolytic activity of MMPs by competing with them (11).

*Serine proteases and their inhibitors* Urokinase-type plasminogen activator (uPA) and tissue-type plasminogen activator (tPA) can modulate immune responses by activating MMPs to alter ECM composition, thus promoting the migration of macrophages and dendritic cells and modulating cytokine activity (12).

*Cathepsin* An increased expression of cathepsin B and its nuclear translocation contribute to proinflammatory responses (13). Cathepsin D, an acidic protease active in intracellular protein breakdown, is significantly overexpressed during aging (14).

#### 4.1.5 Lipid metabolites

Abnormal lipid accumulation induces pro-inflammatory genes activation and senescence phenotype (15). Ni et al. suggested that oxidized lipid mediators may serve as novel components of the SASP (16). Moreover, the levels of cyclooxygenase and its major product, prostaglandin E2 (PGE2), are increased in both replicative and premature senescence (17). Leukotriene D4 plays a role in cellular senescence (18).

#### 4.1.6 Extracellular vesicles

EVs are small vesicles that contain proteins, lipids, and noncoding RNAs (19). An increased EV production is a common feature of senescence and senescent cells (20). Secreted EVs interact with or are internalized by recipient cells to transmit pro-senescence signals between cells and organs, and partially induce immune and inflammatory activation (21, 22).

#### 4.1.7 Others

Additionally, the contributions of small molecules, such as ECM, miRNAs, and ROS, to SASP function remain understudied and may be considered an important future target.

### 4.2 Molecular mechanisms of SASP induction

**Figure 2:**
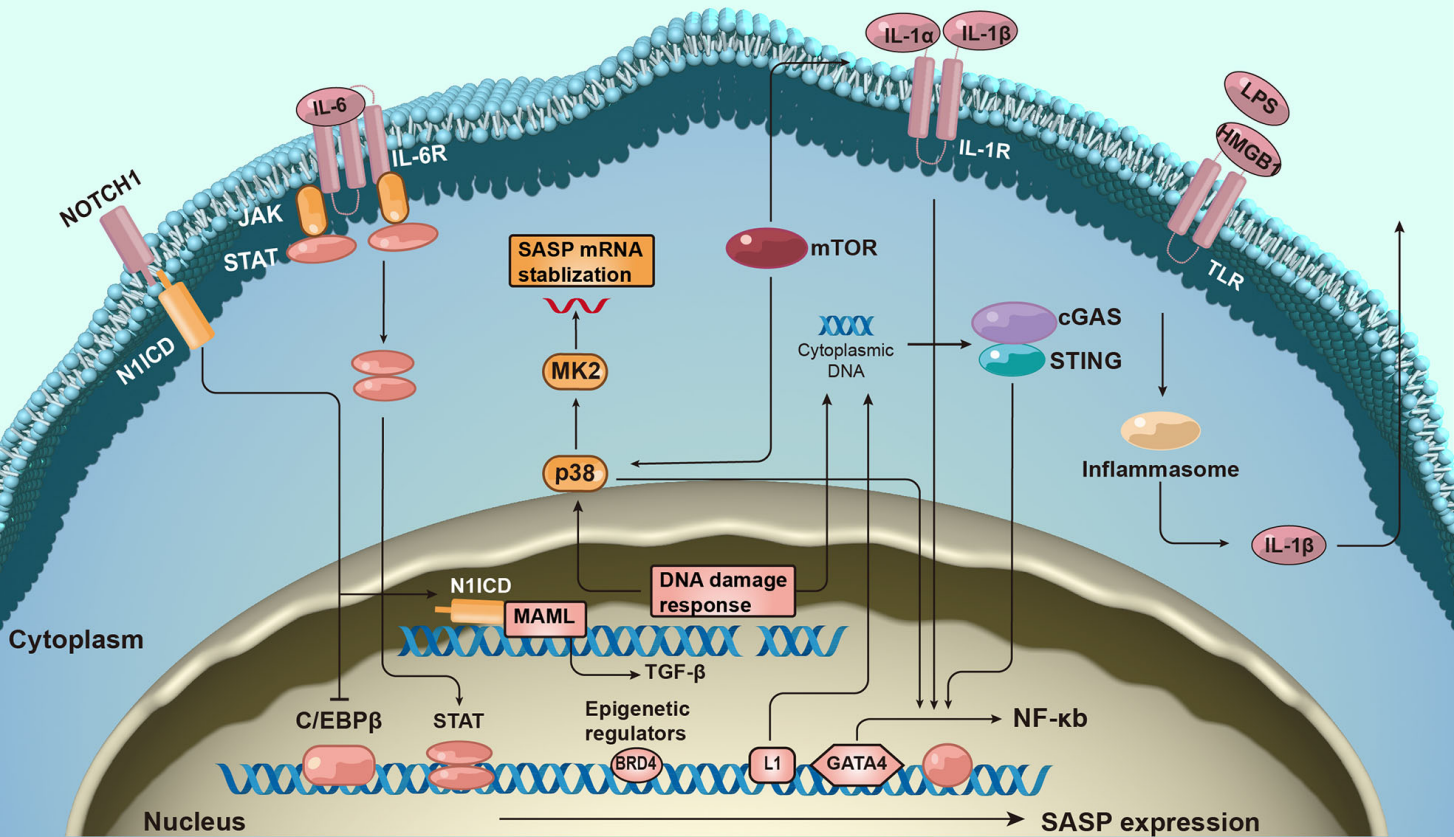
The molecular mechanism of senescence-associated secretory phenotype production. The formation of senescence-associated secretory phenotype (SASP) networks in cells is regulated by a complex molecular mechanism. The DNA damage response is related to senescence activation and SASP expression. SASP protein expression is regulated at both the transcription and post-transcriptional levels. Additionally, epigenetic changes regulate SASP gene expression.

Given the complexity and pleiotropic functionality of the SASP, we generalized the underlying mechanisms regulating it (**Figure 2**). The DNA damage response (DDR) is associated with SASP expression (23). The expression of some inflammatory SASP is regulated by NF-κB and C/EBPβ transcription cofactors by binding to SASP factor promoters. GATA-binding protein 4 transcription factor is responsible for upstream NF-κB signaling and, thus, regulates SASP factor expression (24).

Furthermore, many signaling pathways regulate SASP expression at the transcriptional level. For example, the Janus kinase signal transducer and activator of the transcription pathway participate in regulating SASP expression (25). Activation of p38 signaling also promotes SASP expression (26). More recently, the antiviral cyclic GMP-AMP synthase (cGAS)-stimulator of interferon genes (STING) pathway has been found to be important for SASP expression (27). Additionally, NOTCH signaling regulates the dynamic SASP transition (28).

SASP expression is transcriptionally regulated. mTOR pathway activation promotes the translation of SASP factors such as IL-1α. mTOR also stabilizes SASP mRNA transcripts by regulating MAPKAPK2 translation (29). Inflammasomes are key mediators of SASP induction; inflammasomes upstream of caspase-1 can activate the IL-1 inflammatory cascade during senescence (30).

The expression of SASP also correlates with epigenetic changes. Recruitment of chromatin reader bromodomain-containing protein 4 (BRD4) leads to the remodeling of super-enhancer elements adjacent to SASP genes (31). Several ncRNAs can also affect SASP production at transcriptional and post-translational levels.

### 4.3 Source of oral SASP

#### 4.3.1 Oral senescent cells with locally secreted SASP

*Dental tissue-derived cells* Long-term stress on teeth may induce the human dental pulp cells senescence and up-regulation of SASP factors in human dental pulp cells (32). In aging patients, dental pulp stem cells exhibit elevated expression of SASP factors (33). Dental follicle cells are positive for SA-β-gal staining in later stages of cell culture (34). Dental follicle stem cells secrete TGF-β3, TSP-1, and TGF-β2 to promote and relieve inflammation (35–37).

*Periodontal-derived cells* Senescent periodontal ligament cells express high levels of MMP2 (38). The induction of senescent periodontal ligament stem cell by TGF-β is accompanied by increased levels of certain SASP factors (39). *In vitro* experiments have shown that p16, p21, IL-6, and IL-8 mRNA expression in human gingival fibroblasts is upregulated after replicative senescence (40–42). Researchers have observed that liposaccharide (LPS) exposure causes osteocyte senescence and SASP expression by activating p53 (43).

*Oral mucosa cells* SASP secretion is significantly increased in senescent human oral keratinocytes (44–47), and IL-6, TNF-α, and IFN-γ levels are increased in the oral tongue tissues of the elderly (48). Tongue muscle stem cells and epithelial cells have been shown to degenerate with age, but the relevant SASP profile has not been tested (49, 50).

*Cancer cells* In head and neck squamous cell carcinoma, the number of SA-β-gal-positive aged cells and SASP factor levels are significantly increased after LY2835219 treatment (51). Moreover, an increased secretion of SASP has been observed in senescent cancer-associated fibroblasts (CAF). The senescent CAFs co-cultured with oral squamous cell carcinoma (OSCC) cells also exhibit higher levels of IL-6 and CXCL1 (52). In precancerous lesions, senescent oral submucosal fibroblasts accumulate and upregulate MMPs (53).

*Immunocytes* High-glucose induces macrophage senescence and increases IL-1, IL-6, TNF-α, MMP-2, and MMP-8 secretion (54). Periodontal pathogens induce monocyte activation and the up-regulation of multiple cytokines (55). Overactive neutrophils can release inflammatory molecules and MMPs (56). The SASP profile of B cells and plasma cells in the aging gingival tissue changes (57). The SASP profile varies with cell type; factors inducing senescence are shown in **Table 2**.

**Table 2:** Cellular senescence and SASP involved in the oral cavity.

Cell type		Senescence trigger	SASP factors involved	Ref.
Dental-derived cells	Human dental pulp cells	H_2_O_2_-induced	ICAM-1, VCAM-1, PPAR-g	(58)
	Human dental pulp stem cells	*p*-cresol-induced	IL-6	(33)
	Dental follicle stem cells	LPS-induced	TGF-β2, IL-6, IL-8, IL-1β	(37)
Periodontal -derived cells	Human periodontal ligament fibroblasts	Replicative and radiation-induced	MMP2	(38)
	human periodontal ligament stem cell	TGF-β-induced	IL-8, IL-18, IL-6	(39)
	Human gingival fibroblast	Replicative	IL-6, IL-8	(40)
		Replicative	IL-6, IL-8, TNF, TIMP-1	(41)
		Replicative	MMP3, MMP12, IL-1α	(42)
	Alveolar osteocyte	LPS-induced	ICAM-1, IL-1β, IL-6, IL-8, MCP-1, MMP12, MMP13	(43)
Oral mucosa cells	Human oral keratinocytes	Bisphosphonates-induced	IL-8, IL-6, MMP3	(59)
		Replicative	IL-1β, IL-1α, IL-8, IL-6	(46)
		Replicative	IL-1β, MMP3, PGF, CTGF, VEGF, MMP1, TIMP2, IL-8, MMP9,	(44)
		Replicative	IL-1β, IL-1α, IL-8, IL-6, TNF-α, G-CSF, GM-CSF, GROα	(47)
		High glucose-induced	IL-1β, IL-6, TNF-α	(60)
Cancer cells	Cal27, HSC3 and HSC6 cell lines	LY2835219-induced	IL6, IL8, MCP1, CXCL1, CXCL2, CXCL3	(51)
	CAF from OSCC	Cisplatin-induced	MCP-1, IL-6	(61)
		H_2_O_2_-induced	TGF-β, MMP2	(62)
		Co-culture with OSCC cells	IL-6, CXCL1	(52)
		The progression of oral submucous fibrosis	MMP1, MMP2	(53)
	Oral submucous fibroblasts	High glucose-induced	IL-1, IL-6, TNF-α, MMP-2, and MMP-8	(54)
Immunocytes	Macrophage	*Pg* and *Aa*-induced	IL-1β, TNF-α, IL-6, IL-23	(55)
	Monocytes	LPS, *Pg*, *Aa* and zymosan A-induced	IL-8, MMP-9	(56)
	Neutrophils	Replicative And Periodontal pathogens-induced	MMP2, MMP9, CTSK, TNF-α	(57)
	B cells/plasmacytes	*Pg*-induced	IL-17A, IFN-γ	(63)

CAF, cancer-associated fibroblast; OSCC, oral squamous cell carcinoma; LPS, lipopolysaccharide; ICAM, intercellular adhesion molecule; VCAM, vascular cell adhesion molecule; PPAR, peroxisome proliferator-activated receptor; IL, interleukin; MCP, monocyte chemoattractant protein; MMP, matrix metalloproteinase; TNF, tumor necrosis factor; PGF, placental growth factor; CTGF, connective tissue growth factor; VEGF, vascular endothelial-derived growth factor; TIMP, tissue inhibitor of metalloproteinases; G-CSF, granulocyte colony-stimulating factor; GM-CSF, granulocyte-macrophage colony-stimulating factor; GROα(CXCL1), C-X-C motif chemokine ligand 1; Pg, Porphyromonas gingivalis; Aa, Aggregatibacter actinomycetemcomitans.

#### 4.3.2 ARDs with increased circulating SASP

Circulating SASP is associated with aging and age-related diseases (ARDs) (64). Compared to that in young individuals, the proportion of senescent cells is increased in aging individuals; additionally, the levels of some SASP proteins (age-related SASP) increase significantly (65). Concurrently, the premature cells induced by ARDs can accelerate this process (ARD-related SASP) (66). Age-related versus ARD-related SASP and their effects on oral health are shown in **Table 3**.

**Table 3:** Age-related versus ARD-related circulating SASP and effects on oral health.

Age-related or ARD-related		Study type	Circulating increased SASP factors	Effects on oral health
**Age-related**		Cross-sectional study (65)	IL-6, TNF-α, CCL3, CCL4, GDF15, ACTIVIN A, TNFR1, FAS	1. Recruitment of immune cells: chemokines (CCL3, CCL4) 2. Matrix remodeling: MMPs (MMP9) 3. Fibrosis: growth factors (TGF-β) 4. Senescence re-enforcement: cytokines (IL-6, TNF-α, IL-10)
**ARDs**	Diabetes	Case-control study (67)	IL-6	
		Cohort study (68)	IL-6, TNF-α	
	Cancer	Systematic review (69)	IL-6, TGF-β, IL-10	
	Cardiovascular disease	Case-control study (70)	MMP9	
		Systematic review (71)	IL-6	

ARD, age-related disease; SASP, senescence-associated secretory phenotype; GDF, Growth/differentiation factor; TNFR, tumor necrosis factor receptor; CCL, CC chemokine ligand; TNF, tumor necrosis factor; TGF, transforming growth factor; MMP, matrix metalloproteinase.

### 4.4 Pleiotropic effects of SASP on oral immune homeostasis

**Figure 3:**
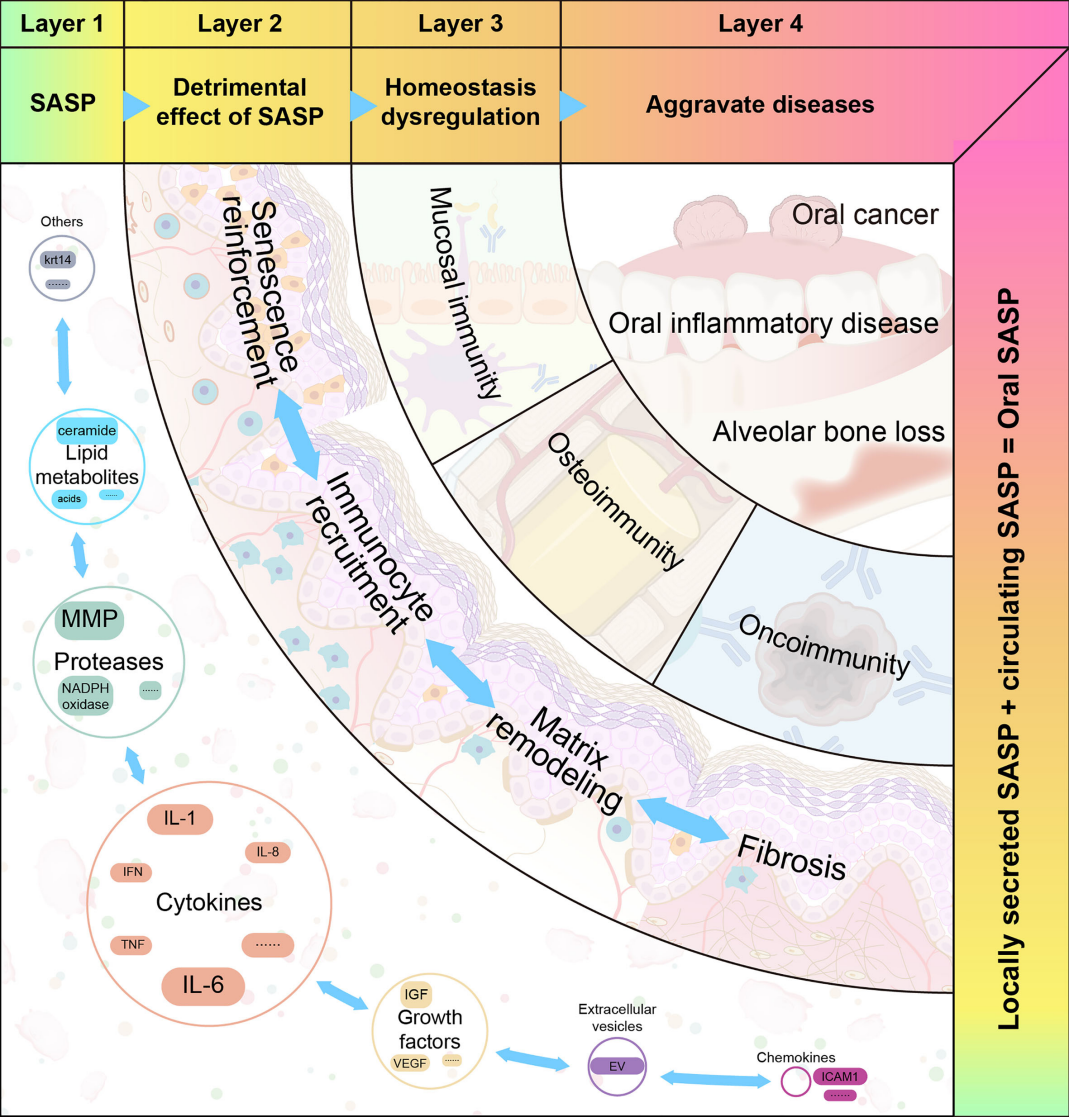
The four proposed layers of how senescence-associated secretory phenotype impacts oral diseases. Senescence-associated secretory phenotype (SASP) (including cytokines, chemokines, growth factors, and proteases) secreted by oral senescent cells, as well as circulating SASP, constitute the aging microenvironment of oral cavity. As an important mediator, SASP accelerates oral pathological alterations including senescence re-enforcement, recruitment of immune cells, matrix remodeling and fibrosis. Then, the SASP-induced dysregulation of immune homeostasis can be divided into three categories: mucosal immunity, bone immunity, and tumor immunity. These destroy the structure and function of different oral tissues. When age-related tissue damage accumulates, it manifests as age-related diseases.

The heterogeneity of SASP may partly account for its pleiotropic effects. Based on the mechanisms of SASP, we propose a four-step hypothetical framework by which oral disease progresses from the pathologic role of SASP to the destabilization of oral immune homeostasis. The pleiotropic effects of the SASP can be interpreted using the model depicted in **Figure 3**.

#### 4.4.1 Step 1: Induction of cellular senescence in oral microenvironment

Replicative- and stress-induced senescence are the main patterns of cellular senescence in the oral microenvironment.

##### 4.4.1.1 Replicative senescence

Serial cultivation of human diploid cells leads to indefinite cell division, which is currently defined as replicative senescence (72). Senescent cells arising from this physiological phenomenon are defined as primary senescent cells (73), which have a series of typical morphologies and biomarker alterations, including DDR (γ-H2AX and p53), cell cycle arrest (p16^INK4A^ and p21^CDKN1A^), anti-apoptotic genes (BCL-proteins), lysosomal content (SA-β-gal), and heterochromatin markers (H3K9me3 and HP1γ) (2, 74).

##### 4.4.1.2 Stress-induced senescence

Due to various stressors, the stress in senescing cells can be classified as secondary senescent cells as follows: 1) DNA damage-induced senescence, which can lead to cellular senescence by inducing DNA damage (35, 75); 2) chemotherapy-induced senescence, in which chemotherapy and anti-resorptive agents have been shown to induce senescence in oral cells (76); 3) oxidative stress–induced senescence whereby H_2_O_2_ treatment increases the positive rate of SA-β-gal staining in human dental pulp cells; 4) oncogene-induced senescence wherein senescence markers are upregulated in oral premalignant lesions (77). 5) epigenetically induced senescence, which is characterized by the blockade of DNA Methyltransferase 1 (DNMT1) and activation of histone acetylation in oral cells (78); and 6) paracrine senescence wherein the SASP produced by primary senescent cells initiates senescence in surrounding cells.

##### 4.4.1.3 Distinguishing senescence and inflammation

**Figure 4:**
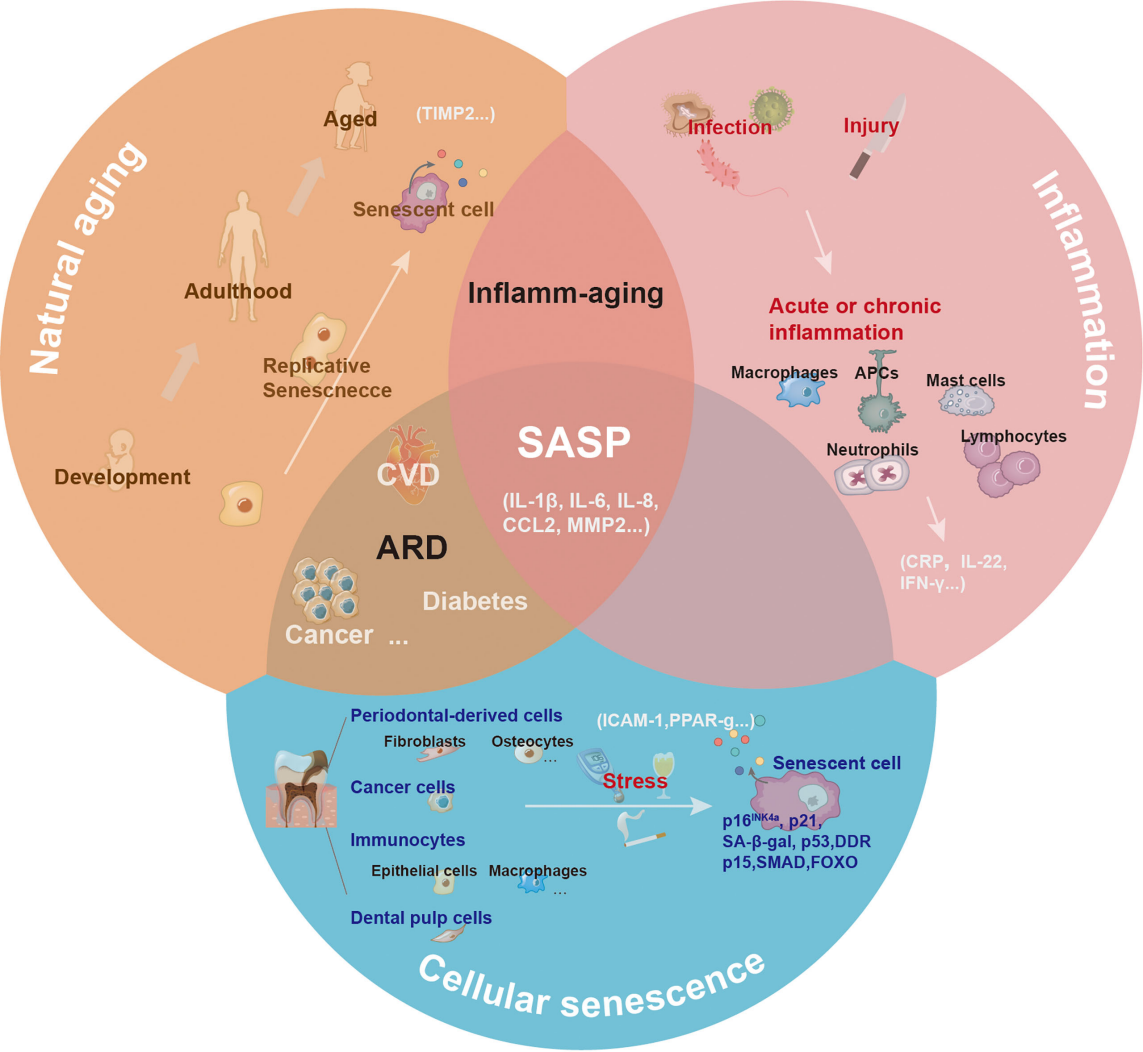
The central role of senescence-associated secretory phenotype in natural aging, inflammation, and cellular senescence. With natural aging, there is a progressive loss in tissue and organ functions, and accumulated senescence-associated secretory phenotype (SASP) can contribute to this process. Depending on the triggers, including infection or injury, the inflammatory response has different pathological consequences. The inflammatory response can be amplified *via* the secretion of inflammatory cytokines. Various cells (such as epithelial cells, dental pulp cells, fibroblasts, and macrophages) in the oral cavity undergoing senescence release SASP to the local microenvironment under the induction of natural aging and stress.

The inflammatory cytokines secreted by activated immune cells overlap with SASP factors. Some cytokines are unique to inflammation (such as IL-22), while others are unique to senescence (such as TIMP). By definition, SASP is downstream of cellular senescence. Notably, the senescence process is generally accompanied by sterile, chronic, low-level inflammation, termed inflamm-aging (79). Chronic inflammation may occur due to age-related immune dysregulation or decreased resistance to challenges, which can induce tissue pathology. The frameworks for aging, inflammation, and cellular senescence are shown in **Figure 4**.

#### 4.4.2 Step 2: SASP accelerates oral pathologic alterations

The SASP is beneficial for maintaining homeostasis and regeneration at a moderate level. However, when SASP is expressed continuously, it induces pathological alterations and disrupts the immune homeostasis of the oral microenvironment.

##### 4.4.2.1 Amplifying the immune cascade

Secreted SASP activates proximal and distant immunocytes in autocrine and paracrine manners. IL-1β is known to induce CD4^+^ T cell proliferation in response to challenges associated with cognate antigens (80). IL-8 induces the migration of activated immune cells to gingival tissue and promotes tissue remodeling and angiogenesis (81). Elevated CCL2 and CCL4 levels are responsible for macrophage recruitment in periodontal lesions (82). The CXCL1 secreted by tumor cells promotes tumor growth by recruiting tumor-associated neutrophils (83).

##### 4.4.2.2 Supporting senescence reinforcement

Certain key SASP factors, such as IL-6, IL-8, GROα, and IGFBP-7, act in an autocrine feedback loop. Non-senescent human oral epithelial keratinocytes cultured with senescent cell supernatants exhibit increased SA-β-gal activity and SASP expression (84). The IL-1β expressed by tumor cells can significantly increase CXCL1 production in CAFs *via* paracrine signaling (85). Moreover, IL-1β induces significant IL-6 production in human gingival fibroblasts and promotes cellular responsiveness to IL-6 through an autocrine loop (86). IL-6 induces normal fibroblast senescence by establishing a senescence induction circuit (87).

##### 4.4.2.3 Remodeling the extracellular matrix

MMPs constitute an important proteolytic pathway that affects tissue remodeling and ECM structure. MMP expression reduces the ability of tissues to maintain homeostasis (88). Specifically, MMP-1 destroys the periodontal connective tissue by directly degrading collagen or activating the fibrinolytic protease cascade, leading to tooth loss (89). The proinflammatory factors IL-1β and IL-6 aggravate tissue destruction by increasing MMP-1 in periodontal tissue (90, 91). Furthermore, TNF-α is important for osteoclast formation and bone resorption in mice and suppresses ECM protein expression (92, 93).

##### 4.4.2.4 Promoting fibrosis

TGF-β is the primary factor driving fibrosis. TGF-β activation in epithelial cells can interact with fibroblast cells and induce the expression of other profibrotic cytokines (e.g., endothelin and CTGF) (94). Areca nut alkaloids induce senescence in oral fibroblasts and TGF-β production, which is favorable for the development of oral submucosal fibrosis (95). TIMP-1 and -2 have also been proven to be early indicators of oral submucous fibrosis and aging (96). Additionally, the MMP-1 and MMP-3 secreted by senescent cells during oral submucosal fibrosis have been shown to promote fibrosis in the advanced stages (97).

#### 4.4.3 Step 3: SASP disrupts oral immune homeostasis

##### 4.4.3.1 Effects on mucosal immunity

SASP challenges mucosal epithelial homeostasis by undermining the physical barrier. MMP-2 cleaves cell–cell adhesion molecules, thus disrupting epithelial adhesion (62). An increased MMP-1 expression in inflamed tissues directly leads to the degradation of collagen, thereby causing tissue destruction (89). TNF modulates the apoptosis of epithelial cells and fibroblasts and suppresses ECM proteins. These results indicate that TNF—in the senescence process—can damage the epithelial barrier (93). Some SASP factors participate in the recruitment and activation of immune cells. IL-1β greatly induces the proliferation and activation of Th1 and Th2 cells (80). IL-8 regulates neutrophil activation and migration in inflamed tissues (98). Additionally, IL-6 significantly increases the production of VEGF, bFGF, and cathepsin B in human gingival fibroblasts and synergistically induces angiogenesis in periodontitis lesions (86, 99). Senescent macrophages in the gingiva contribute to SASP release and inflammatory response, which indicates that senescence may also play an important role.

##### 4.4.3.2 Effect on osteoimmunity

The secretome produced *via* innate host responses facilitates communication between immune cells and bone cells. Senescent immune cells regulate bone homeostasis through immune mediators that involve the SASP. For instance, IL-17, IL-1, and IL-6, as well as low levels of IFN-γ secreted by Th17 cells, promote osteoclastogenesis (100, 101). TNF-α has also been shown to strengthen osteoclastogenesis by synergizing with RANKL (102). In contrast, the bone-senescent microenvironment further enhances alveolar bone ageing. SASP factors released extracellularly from osteocytes accelerate the senescence of bone marrow (BM) (103). Selected SASP markers secreted by senescent osteocytes from alveolar bones promote inflammation and alveolar bone loss (43). In senescent fibroblasts, IL-1β increases the production of chemokines, including PGE2, an important chemical mediator of alveolar bone resorption (104). Additionally, senescent osteocytes develop a unique SASP signature composed of upregulated MMPs (105). MMPs can degrade ECM proteins, including sulfated proteoglycans, collagen, and fibronectin, in cartilage. Moreover, insulin-like growth factor-binding protein 4 (IGFBP-4) are upregulated in senescent osteocytes and myeloid cells, leading to deficiency in bone formation (22, 106).

##### 4.4.3.3 Effect on oncoimmunity

Senescent cells in the tumor microenvironment (TME) may play roles in tumor progression and metastasis. CAFs are the most prominent stromal cells in TME. CAFs are senescent cells that actively communicate with other cells in the TME by secreting the SASP. TGF-β levels are upregulated by senescent oral CAFs and synergize with MMP-2 to reduce the expression of cell adhesion molecules and promote epithelial invasion (107). CAFs also modulate the epithelial-mesenchymal transition (EMT) by secreting TGF-β (108). Moreover, activated CAFs secrete proinflammatory factors that recruit and activate infiltrating immune cells (IICs). IICs provide mitogenic growth factors that stimulate the proliferation of tumor cells and other nearby stromal cells (109). IICs also express multiple proteolytic enzymes that selectively modify ECM structure and composition (110). Additionally, Park et al. proposed that the serum levels of IL-6 may be a serum biomarker for OSCC diagnosis (111). IL-6 promotes the invasion of cancer cells through the epithelial–mesenchymal transition (112). However, the immune cell subtype and its mechanism in the TME require further elucidation; further research is necessary to determine the specific roles of these factors in oral cancer.

#### 4.4.4 Step 4: Aging and SASP in oral diseases

##### 4.4.4.1 SASP in oral inflammatory disease

The SASP may be responsible for chronic oral inflammation, as it disrupts mucosal homeostasis through matrix degradation, senescence reinforcement, and immune cell recruitment. Compared to their young counterparts, old mice suffer frequent spontaneous periodontitis, and the expression of IL-1β and TNF-α in the gingiva is significantly elevated (113). Increased levels of IL-6 and MMP-8 have been observed in the saliva of patients with chronic periodontitis (114). Enhanced senescence and increased SASP are observed after ligation and *P. gingivalis* infection–induced periodontitis *in vivo* (115, 116). Additionally, hyperglycemia can increase the burden of senescence in the gingival tissue (54). Senescent cells accumulate in aged and diseased oral tissues, and this accumulation is associated with severe tissue destruction.

##### 4.4.4.2 SASP in alveolar bone loss

Bone integrity and quality undergo differential changes in various oral diseases (117). Animal studies have shown that aging is positively correlated with alveolar bone loss. Old mice have poorer alveolar bone quality, lower alveolar bone crest height, and more active bone resorption (118). Senescence-associated distension of satellites (an early and consistent marker of senescence) and p16 mRNA expression are increased in old alveolar bone samples (119). Moreover, senescent osteocytes show changes in cell phenotype and diminished osteocyte density during age-related skeletal changes. This may further damage the mechanical conduction, impair nutrient access, influence signal transduction, and ultimately result in significant bone loss (120). Senescent bone cells exacerbate chronic inflammation through SASP accumulation, leading to deterioration of the periodontal environment (119). The SASP factor secreted by LPS-induced senescent osteocytes promotes the proliferation of some oral pathogens. These pathogens produce more LPS, thereby exacerbating the senescence of alveolar osteocytes and resulting in alveolar bone loss (43).

##### 4.4.4.3 SASP in oral cancer

Cell senescence occurs throughout life and plays dual roles in modulating the progression and suppression of oral cancers (121). The number of SA-β-Gal-positive cells is higher in OSCC specimens than in tumor-free marginal tissues (52). Senescent fibroblasts also accumulate in precancerous lesions *in vivo* (53). Senescent cells secrete many SASP factors into the TME, which may support cell proliferation, EMT, and angiogenesis, thereby promoting tumor growth and invasion. MMP-1, -2, -10, and-12 levels in the saliva of OSCC patients increase significantly (122). In OSCC, the expression of MMP-11 is associated with an increased lymph node metastasis and a low survival rate (123). MMP-7 is mainly expressed in the invasive portion of oral cancer, whereas MMP-8 and MMP-9 are mainly detected in peritumoral inflammatory cells (124). This evidence suggests that senescent cells and the SASP are key factors in the onset and progression of oral cancer.

## 5 Concluding remarks

SASP, derived from senescent cells, includes secreted factors that may alter the extracellular environment (proteases), mediators that transmit and amplify senescence signals (cytokines, chemokines, bioactive lipids, and EVs), and proteins that influence cancer behavior (growth factors). The composition of SASP in the oral environment consists of two parts: local SASP and circulating SASP. Local SASP is secreted by oral senescent cells undergoing primary or secondary senescent patterns while the circulating SASP is closely associated with chronological age and ARD. As an important bridge for intercellular communication, the SASP communicates with different immune cells and is the key to securing oral homeostasis. Conversely, the SASP-induced dysregulation of immune homeostasis leads to intrinsically complex phenotypes in oral pathology. A better understanding of the relationship between SASP and the immune system is necessary for developing therapies to prevent or treat various ARDs in the oral cavity.

## Author contributions

ZY and LN contributed to design, drafted and revised the manuscript; PZ contributed to design, and critically revised the manuscript; NJ and GL. assisted data analysis. QW, contributed to conception, design, and critically revised the manuscript. All authors gave final approval and agree to be accountable for all aspects of the work.

## Funding

The authors disclosed receipt of the following financial support for the research, authorship, and/or publication of this article: this study was supported by the National Natural Science Foundation of China (81870779) and Sichuan Provincial Science and Technology Innovation Talent Program (2021JDRC0036). Chengdu Science & Technology Program (2022-YF05-01760-SN).

## Conflict of interest

The authors declare that the research was conducted in the absence of any commercial or financial relationships that could be construed as a potential conflict of interest.

## Publisher’s note

All claims expressed in this article are solely those of the authors and do not necessarily represent those of their affiliated organizations, or those of the publisher, the editors and the reviewers. Any product that may be evaluated in this article, or claim that may be made by its manufacturer, is not guaranteed or endorsed by the publisher.

## References

[1] Zubeidat K Hovav A-H . Shaped by the epithelium-postnatal immune mechanisms of oral homeostasis. Trends Immunol (2021) 42:622–34. doi: 10.1016/j.it.2021.05.006 34083119

[2] Calcinotto A Kohli J Zagato E Pellegrini L Demaria M Alimonti A . Cellular senescence: Aging, cancer, and injury. Physiol Rev (2019) 99:1047–78. doi: 10.1152/physrev.00020.2018 30648461

[3] Basisty N Kale A Jeon OH Kuehnemann C Payne T Rao C . A proteomic atlas of senescence-associated secretomes for aging biomarker development. PLoS Biol (2020) 18:e3000599–e3000599. doi: 10.1371/journal.pbio.3000599 31945054 PMC6964821

[4] Hernandez-Segura A De Jong TV Melov S Guryev V Campisi J Demaria M . Unmasking transcriptional heterogeneity in senescent cells. Curr Biol (2017) 27:2652–60.e4. doi: 10.1016/j.cub.2017.07.033 28844647 PMC5788810

[5] Gardner SE Humphry M Bennett MR Clarke MCH . Senescent vascular smooth muscle cells drive inflammation through an interleukin-1α-Dependent senescence-associated secretory phenotype. Arteriosclerosis thrombosis Vasc Biol (2015) 35:1963–74. doi: 10.1161/ATVBAHA.115.305896 PMC4548545 26139463

[6] Migliorini P Italiani P Pratesi F Puxeddu I Boraschi D . The IL-1 family cytokines and receptors in autoimmune diseases. Autoimmun Rev (2020) 19:102617. doi: 10.1016/j.autrev.2020.102617 32663626

[7] Schaper F Rose-John S . Interleukin-6: Biology, signaling and strategies of blockade. Cytokine Growth Factor Rev (2015) 26:475–87. doi: 10.1016/j.cytogfr.2015.07.004 26189695

[8] Varfolomeev E Vucic D . Intracellular regulation of TNF activity in health and disease. Cytokine (2018) 101:26–32. doi: 10.1016/j.cyto.2016.08.035 27623350

[9] Sokol CL Luster AD . The chemokine system in innate immunity. Cold Spring Harbor Perspect Biol (2015) 7:a016303. doi: 10.1101/cshperspect.a016303 PMC4448619 25635046

[10] Smith PC Martínez C Cáceres M Martínez J . Research on growth factors in periodontology. Periodontology 2000 (2015) 67:234–50. doi: 10.1111/prd.12068 25494603

[11] Checchi V Maravic T Bellini P Generali L Consolo U Breschi L . The role of matrix metalloproteinases in periodontal disease. Int J Environ Res Public Health (2020) 17 (14):4923. doi: 10.3390/ijerph17144923 PMC7399864 32650590

[12] Gonias SL . Plasminogen activator receptor assemblies in cell signaling, innate immunity, and inflammation. Am J Physiology-Cell Physiol (2021) 321:C721–34. doi: 10.1152/ajpcell.00269.2021 PMC8560384 34406905

[13] Meng J Liu Y Xie Z Qing H Lei P Ni J . Nucleus distribution of cathepsin b in senescent microglia promotes brain aging through degradation of sirtuins. Neurobiol Aging (2020) 96:255–66. doi: 10.1016/j.neurobiolaging.2020.09.001 33049518

[14] Byun H-O Han N-K Lee H-J Kim K-B Ko Y-G Yoon G . Cathepsin d and eukaryotic translation elongation factor 1 as promising markers of cellular senescence. Cancer Res (2009) 69:4638–47. doi: 10.1158/0008-5472.CAN-08-4042 19487283

[15] Hamsanathan S Gurkar AU . Lipids as regulators of cellular senescence. Front Physiol (2022) 13:796850–0. doi: 10.3389/fphys.2022.796850 PMC8965560 35370799

[16] Ni C Narzt M-S Nagelreiter I-M Zhang CF Larue L Rossiter H . Autophagy deficient melanocytes display a senescence associated secretory phenotype that includes oxidized lipid mediators. Int J Biochem Cell Biol (2016) 81:375–82. doi: 10.1016/j.biocel.2016.10.006 27732890

[17] Wu D Mura C Beharka AA Han SN Paulson KE Hwang D . Age-associated increase in PGE2 synthesis and COX activity in murine macrophages is reversed by vitamin e. Am J Physiology-Cell Physiol (1998) 275:C661–8. doi: 10.1152/ajpcell.1998.275.3.C661 9730949

[18] Wei J Chen S Guo W Feng B Yang S Huang C . Leukotriene D4 induces cellular senescence in osteoblasts. Int Immunopharmacol (2018) 58:154–9. doi: 10.1016/j.intimp.2017.12.027 29587204

[19] Yin Y Chen H Wang Y Zhang L Wang X . Roles of extracellular vesicles in the aging microenvironment and age-related diseases. J extracellular vesicles (2021) 10:e12154–4. doi: 10.1002/jev2.12154 PMC8491204 34609061

[20] Robbins PD . Extracellular vesicles and aging. Stem Cell Invest (2017) 4:98–8. doi: 10.21037/sci.2017.12.03 PMC5762993 29359137

[21] Gonda A Kabagwira J Senthil GN Wall NR . Internalization of exosomes through receptor-mediated endocytosis. Mol Cancer Res (2019) 17:337–47. doi: 10.1158/1541-7786.MCR-18-0891 30487244

[22] Al Suraih MS Trussoni CE Splinter PL Larusso NF O'hara SP . Senescent cholangiocytes release extracellular vesicles that alter target cell phenotype *via* the epidermal growth factor receptor. Liver Int (2020) 40:2455–68. doi: 10.1111/liv.14569 PMC7669612 32558183

[23] Rodier F Coppé JP Patil CK Hoeijmakers WA Muñoz DP Raza SR . Persistent DNA damage signalling triggers senescence-associated inflammatory cytokine secretion. Nat Cell Biol (2009) 11:973–9. doi: 10.1038/ncb1909 PMC2743561 19597488

[24] Kang C Xu Q Martin TD Li MZ Demaria M Aron L . The DNA damage response induces inflammation and senescence by inhibiting autophagy of GATA4. Science (2015) 349:aaa5612. doi: 10.1126/science.aaa5612 26404840 PMC4942138

[25] Farr JN Xu M Weivoda MM Monroe DG Fraser DG Onken JL . Targeting cellular senescence prevents age-related bone loss in mice. Nat Med (2017) 23:1072–9. doi: 10.1038/nm.4385 PMC5657592 28825716

[26] Freund A Patil CK Campisi J . p38MAPK is a novel DNA damage response-independent regulator of the senescence-associated secretory phenotype. EMBO J (2011) 30:1536–48. doi: 10.1038/emboj.2011.69 PMC3102277 21399611

[27] De Cecco M Ito T Petrashen AP Elias AE Skvir NJ Criscione SW . L1 drives IFN in senescent cells and promotes age-associated inflammation. Nature (2019) 566:73–8. doi: 10.1038/s41586-018-0784-9 PMC6519963 30728521

[28] Ito Y Hoare M Narita M . Spatial and temporal control of senescence. Trends Cell Biol (2017) 27:820–32. doi: 10.1016/j.tcb.2017.07.004 28822679

[29] Herranz N Gallage S Mellone M Wuestefeld T Klotz S Hanley CJ . mTOR regulates MAPKAPK2 translation to control the senescence-associated secretory phenotype. Nat Cell Biol (2015) 17:1205–17. doi: 10.1038/ncb3225 PMC4589897 26280535

[30] He M Chiang HH Luo H Zheng Z Qiao Q Wang L . An acetylation switch of the NLRP3 inflammasome regulates aging-associated chronic inflammation and insulin resistance. Cell Metab (2020) 31:580–91.e5. doi: 10.1016/j.cmet.2020.01.009 32032542 PMC7104778

[31] Tasdemir N Banito A Roe JS Alonso-Curbelo D Camiolo M Tschaharganeh DF . BRD4 connects enhancer remodeling to senescence immune surveillance. Cancer Discovery (2016) 6:612–29. doi: 10.1158/2159-8290.CD-16-0217 PMC4893996 27099234

[32] Maeda H . Aging and senescence of dental pulp and hard tissues of the tooth. Front Cell Dev Biol (2020) 8:605996. doi: 10.3389/fcell.2020.605996 33330507 PMC7734349

[33] Zayed M Iohara K . Effects of p-cresol on senescence, survival, inflammation, and odontoblast differentiation in canine dental pulp stem cells. Int J Mol Sci (2020) 21 (18) :6931. doi: 10.3390/ijms21186931 PMC7555360 32967298

[34] Morsczeck C . Effects of cellular senescence on dental follicle cells. Pharmacology (2021) 106:137–42. doi: 10.1159/000510014 PMC8120660 32980839

[35] Zhai Y Wei R Liu J Wang H Cai W Zhao M . Drug-induced premature senescence model in human dental follicle stem cells. Oncotarget (2017) 8:7276–93. doi: 10.18632/oncotarget.14085 PMC5352320 28030852

[36] Chen X Yang B Tian J Hong H Du Y Li K . Dental follicle stem cells ameliorate lipopolysaccharide-induced inflammation by secreting TGF-β3 and TSP-1 to elicit macrophage M2 polarization. Cell Physiol Biochem (2018) 51:2290–308. doi: 10.1159/000495873 30537736

[37] Um S Lee J-H Seo B-M . TGF-β2 downregulates osteogenesis under inflammatory conditions in dental follicle stem cells. Int J Oral Sci (2018) 10:29–9. doi: 10.1038/s41368-018-0028-8 PMC6175959 30297828

[38] Konstantonis D Papadopoulou A Makou M Eliades T Basdra EK Kletsas D . Senescent human periodontal ligament fibroblasts after replicative exhaustion or ionizing radiation have a decreased capacity towards osteoblastic differentiation. Biogerontology (2013) 14:741–51. doi: 10.1007/s10522-013-9449-0 23934584

[39] Fan C Ji Q Zhang C Xu S Sun H Li Z . TGF−β induces periodontal ligament stem cell senescence through increase of ROS production. Mol Med Rep (2019) 20 (4):3123–30. doi: 10.3892/mmr.2019.10580 PMC6755147 31432132

[40] Xia Y Sun M Xie Y Shu R . mTOR inhibition rejuvenates the aging gingival fibroblasts through alleviating oxidative stress. Oxid Med Cell Longev (2017) 2017:6292630. doi: 10.1155/2017/6292630 28804534 PMC5540269

[41] Páez J Hernández R ESPINOZA J ROJAS L Martínez CE TOBAR N . Uncoupled inflammatory, proliferative, and cytoskeletal responses in senescent human gingival fibroblasts. J Periodontal Res (2020) 55:432–40. doi: 10.1111/jre.12727 31943227

[42] Kim S Ahn SH Lee J-S Song J-E Cho S-H Jung S . Differential matrix metalloprotease (MMP) expression profiles found in aged gingiva. PLoS One (2016) 11:e0158777–e0158777. doi: 10.1371/journal.pone.0158777 27391467 PMC4938517

[43] Aquino-Martinez R Rowsey JL Fraser DG Eckhardt BA Khosla S Farr JN . LPS-induced premature osteocyte senescence: Implications in inflammatory alveolar bone loss and periodontal disease pathogenesis. Bone (2020) 132:115220. doi: 10.1016/j.bone.2019.115220 31904537 PMC6990876

[44] Jang DH Bhawal UK Min HK Kang HK Abiko Y Min BM . A transcriptional roadmap to the senescence and differentiation of human oral keratinocytes. J Gerontol A Biol Sci Med Sci (2015) 70:20–32. doi: 10.1093/gerona/glt212 24398559

[45] Zhao P Yue Z Nie L Zhao Z Wang Q Chen J . Hyperglycaemia-associated macrophage pyroptosis accelerates periodontal inflamm-aging. J Clin Periodontology (2021) 48:1379–92. doi: 10.1111/jcpe.13517 34219262

[46] Niklander S Bandaru D Lambert DW Hunter KD . ROCK inhibition modulates the senescence-associated secretory phenotype (SASP) in oral keratinocytes. FEBS Open Bio (2020) 10:2740–9. doi: 10.1002/2211-5463.13012 PMC7714064 33095981

[47] Schwartz RE Shokhirev MN Andrade LR Gutkind JS Iglesias-Bartolome R Shadel GS . Insights into epithelial cell senescence from transcriptome and secretome analysis of human oral keratinocytes. Aging (2021) 13:4747–77. doi: 10.18632/aging.202658 PMC7950289 33601339

[48] Bhaskaran N Faddoul F Paes Da Silva A Jayaraman S Schneider E Mamileti P . IL-1β-MyD88-mTOR axis promotes immune-protective IL-17A(+)Foxp3(+) cells during mucosal infection and is dysregulated with aging. Front Immunol (2020) 11:595936–6. doi: 10.3389/fimmu.2020.595936 PMC7677307 33240286

[49] Kletzien H Kelm-Nelson CA Wang S Suzuki M Connor NP . Myogenic marker expression as a function of age and exercise-based therapy in the tongue. Exp gerontology (2020) 142:111104–4. doi: 10.1016/j.exger.2020.111104 PMC7748063 33017670

[50] Barlagiannis D Dietrich E Papaliagkas V Makri S Toskas A Papamitsou T . Ultrastructural aspects of the effects of l-carnitine administration on epithelial cells in the aging rat tongue. Hippokratia (2014) 18 (1):32–6. PMC4103038 25125949

[51] Hu Q Peng J Jiang L Li W Su Q Zhang J . Metformin as a senostatic drug enhances the anticancer efficacy of CDK4/6 inhibitor in head and neck squamous cell carcinoma. Cell Death Dis (2020) 11:925. doi: 10.1038/s41419-020-03126-0 33116117 PMC7595194

[52] Kim EK Moon S Kim DK Zhang X Kim J . CXCL1 induces senescence of cancer-associated fibroblasts *via* autocrine loops in oral squamous cell carcinoma. PLoS One (2018) 13:e0188847. doi: 10.1371/journal.pone.0188847 29360827 PMC5779641

[53] Pitiyage GN Slijepcevic P Gabrani A Chianea YG Lim KP Prime SS . Senescent mesenchymal cells accumulate in human fibrosis by a telomere-independent mechanism and ameliorate fibrosis through matrix metalloproteinases. J Pathol (2011) 223:604–17. doi: 10.1002/path.2839 21341274

[54] Zhang P Wang Q Nie L Zhu R Zhou X Zhao P . Hyperglycemia-induced inflamm-aging accelerates gingival senescence *via* NLRC4 phosphorylation. J Biol Chem (2019) 294:18807–19. doi: 10.1074/jbc.RA119.010648 PMC6901307 31676687

[55] Cheng W-C Van Asten SD Burns LA Evans HG Walter GJ Hashim A Hughes FJ . Periodontitis-associated pathogens p. gingivalis and a. actinomycetemcomitans activate human CD14+ monocytes leading to enhanced Th17/IL-17 responses. Eur J Immunol (2016) 46:2211–21. doi: 10.1002/eji.201545871 PMC5031191 27334899

[56] Restaíno CG Chaparro A Valenzuela MA Kettlun AM Vernal R Silva A . Stimulatory response of neutrophils from periodontitis patients with periodontal pathogens. Oral Dis (2007) 13:474–81. doi: 10.1111/j.1601-0825.2006.01323.x 17714350

[57] Ebersole JL Kirakodu SS Novak MJ Orraca L Martinez JG Cunningham LL . Transcriptome analysis of b cell immune functions in periodontitis: Mucosal tissue responses to the oral microbiome in aging. Front Immunol (2016) 7:272–2. doi: 10.3389/fimmu.2016.00272 PMC4947588 27486459

[58] Lee Y-H Kim G-E Cho H-J Yu M-K Bhattarai G Lee N-H . Aging of *In vitro* pulp illustrates change of inflammation and dentinogenesis. J Endodontics (2013) 39:340–5. doi: 10.1016/j.joen.2012.10.031 23402504

[59] Kim RH Lee RS Williams D Bae S Woo J Lieberman M . Bisphosphonates induce senescence in normal human oral keratinocytes. J Dent Res (2011) 90:810–6. doi: 10.1177/0022034511402995 PMC3144120 21427353

[60] Zhang P Lu B Zhu R Yang D Liu W Wang Q . Hyperglycemia accelerates inflammaging in the gingival epithelium through inflammasomes activation. J Periodontal Res (2021) 56 (4):667–78. doi: 10.1111/jre.12863 33650689

[61] Kabir TD Leigh RJ Tasena H Mellone M Coletta RD Parkinson EK . A miR-335/COX-2/PTEN axis regulates the secretory phenotype of senescent cancer-associated fibroblasts. Aging (Albany NY) (2016) 8:1608–35. doi: 10.18632/aging.100987 PMC5032686 27385366

[62] Hassona Y Cirillo N Heesom K Parkinson EK Prime SS . Senescent cancer-associated fibroblasts secrete active MMP-2 that promotes keratinocyte dis-cohesion and invasion. Br J Cancer (2014) 111:1230–7. doi: 10.1038/bjc.2014.438 PMC4453858 25117810

[63] Cheng W-C Hughes FJ Taams LS . The presence, function and regulation of IL-17 and Th17 cells in periodontitis. J Clin Periodontology (2014) 41:541–9. doi: 10.1111/jcpe.12238 24735470

[64] He S Sharpless NE . Senescence in health and disease. Cell (2017) 169:1000–11. doi: 10.1016/j.cell.2017.05.015 PMC5643029 28575665

[65] Schafer MJ Zhang X Kumar A Atkinson EJ Zhu Y Jachim S . The senescence-associated secretome as an indicator of age and medical risk. JCI Insight (2020) 5 (12):e133668 doi: 10.1172/jci.insight.133668 PMC7406245 32554926

[66] Wang Q Nie L Zhao P Zhou X Ding Y Chen Q . Diabetes fuels periodontal lesions *via* GLUT1-driven macrophage inflammaging. Int J Oral Sci (2021) 13:11. doi: 10.1038/s41368-021-00116-6 33762572 PMC7990943

[67] Pradhan AD Manson JE Rifai N Buring JE Ridker PM . C-reactive protein, interleukin 6, and risk of developing type 2 diabetes mellitus. JAMA (2001) 286:327–34. doi: 10.1001/jama.286.3.327 11466099

[68] Spranger J Kroke A MöHlig M Hoffmann K Bergmann MM Ristow M . Inflammatory cytokines and the risk to develop type 2 diabetes: Results of the prospective population-based European prospective investigation into cancer and nutrition (EPIC)-potsdam study. Diabetes (2003) 52:812–7. doi: 10.2337/diabetes.52.3.812 12606524

[69] Lippitz BE . Cytokine patterns in patients with cancer: a systematic review. Lancet Oncol (2013) 14:e218–28. doi: 10.1016/S1470-2045(12)70582-X 23639322

[70] Ferroni P Basili S Martini F Cardarello CM Ceci F Franco MD . Serum metalloproteinase 9 levels in patients with coronary artery disease: A novel marker of inflammation. J Invest Med (2003) 51:295. doi: 10.1136/jim-51-05-17 14577520

[71] Zhang B Li XL Zhao CR Pan CL Zhang Z . Interleukin-6 as a predictor of the risk of cardiovascular disease: A meta-analysis of prospective epidemiological studies. Immunol Invest (2018) 47:689–99. doi: 10.1080/08820139.2018.1480034 29873573

[72] Hayflick L Moorhead PS . The serial cultivation of human diploid cell strains. Exp Cell Res (1961) 25:585–621. doi: 10.1016/0014-4827(61)90192-6 13905658

[73] Ebersole JL Graves CL Gonzalez OA Dawson D 3rd Morford LA Huja PE . Aging, inflammation, immunity and periodontal disease. Periodontol 2000 (2016) 72:54–75. doi: 10.1111/prd.12135 27501491

[74] Hernandez-Segura A Nehme J Demaria M . Hallmarks of cellular senescence. Trends Cell Biol (2018) 28:436–53. doi: 10.1016/j.tcb.2018.02.001 29477613

[75] Hodjat M Rezvanfar MA Abdollahi M . A systematic review on the role of environmental toxicants in stem cells aging. Food Chem Toxicol (2015) 86:298–308. doi: 10.1016/j.fct.2015.11.002 26582272

[76] Childs BG Li H Van Deursen JM . Senescent cells: a therapeutic target for cardiovascular disease. J Clin Invest (2018) 128:1217–28. doi: 10.1172/JCI95146 PMC5873883 29608141

[77] Bascones-Martínez A López-Durán M Cano-Sánchez J Sánchez-Verde L Díez-Rodríguez A Aguirre-Echebarría P . Differences in the expression of five senescence markers in oral cancer, oral leukoplakia and control samples in humans. Oncol Lett (2012) 3:1319–25. doi: 10.3892/ol.2012.649 PMC3392562 22783442

[78] Hodjat M Khan F Saadat KASM . Epigenetic alterations in aging tooth and the reprogramming potential. Ageing Res Rev (2020) 63:101140. doi: 10.1016/j.arr.2020.101140 32795505

[79] Chung HY Cesari M Anton S Marzetti E Giovannini S Seo AY . Molecular inflammation: underpinnings of aging and age-related diseases. Ageing Res Rev (2009) 8:18–30. doi: 10.1016/j.arr.2008.07.002 18692159 PMC3782993

[80] Ben-Sasson SZ Hu-Li J Quiel J Cauchetaux S Ratner M Shapira I . IL-1 acts directly on CD4 T cells to enhance their antigen-driven expansion and differentiation. Proc Natl Acad Sci United States America (2009) 106:7119–24. doi: 10.1073/pnas.0902745106 PMC2678417 19359475

[81] Lagdive SS Marawar PP Byakod G Lagdive SB . Evaluation and comparison of interleukin-8 (IL-8) level in gingival crevicular fluid in health and severity of periodontal disease: a clinico-biochemical study. Indian J Dent Res (2013) 24:188–92. doi: 10.4103/0970-9290.116675 23965444

[82] Hou KL Lin SK Kok SH Wang HW Lai EH Hong CY . Increased expression of glutaminase in osteoblasts promotes macrophage recruitment in periapical lesions. J Endod (2017) 43:602–8. doi: 10.1016/j.joen.2016.11.005 28190586

[83] Yuan M Zhu H Xu J Zheng Y Cao X Liu Q . Tumor-derived CXCL1 promotes lung cancer growth *via* recruitment of tumor-associated neutrophils. J Immunol Res (2016) 2016:6530410. doi: 10.1155/2016/6530410 27446967 PMC4942661

[84] Wang H Wang Z Huang Y Zhou Y Sheng X Jiang Q . Senolytics (DQ) mitigates radiation ulcers by removing senescent cells. Front Oncol (2019) 9 1576. doi: 10.3389/fonc.2019.01576 32117790 PMC7034035

[85] Wei LY Lee JJ Yeh CY Yang CJ Kok SH Ko JY . Reciprocal activation of cancer-associated fibroblasts and oral squamous carcinoma cells through CXCL1. Oral Oncol (2019) 88:115–23. doi: 10.1016/j.oraloncology.2018.11.002 30616781

[86] Sawada S Chosa N Ishisaki A Naruishi K . Enhancement of gingival inflammation induced by synergism of IL-1β and IL-6. Biomed Res (2013) 34:31–40. doi: 10.2220/biomedres.34.31 23428978

[87] Kojima H Kunimoto H Inoue T Nakajima K . The STAT3-IGFBP5 axis is critical for IL-6/gp130-induced premature senescence in human fibroblasts. Cell Cycle (2012) 11:730–9. doi: 10.4161/cc.11.4.19172 22374671

[88] Bonnema DD Webb CS Pennington WR Stroud RE Leonardi AE Clark LL . Effects of age on plasma matrix metalloproteinases (MMPs) and tissue inhibitor of metalloproteinases (TIMPs). J Card Fail (2007) 13:530–40. doi: 10.1016/j.cardfail.2007.04.010 PMC2698433 17826643

[89] Emingil G Han B Gürkan A Berdeli A Tervahartiala T Salo T . Matrix metalloproteinase (MMP)-8 and tissue inhibitor of MMP-1 (TIMP-1) gene polymorphisms in generalized aggressive periodontitis: Gingival crevicular fluid MMP-8 and TIMP-1 levels and outcome of periodontal therapy. J Periodontology (2014) 85:1070–80. doi: 10.1902/jop.2013.130365 24283658

[90] Kida Y Kobayashi M Suzuki T Takeshita A Okamatsu Y Hanazawa S . Interleukin-1 stimulates cytokines, prostaglandin E2 and matrix metalloproteinase-1 production *via* activation of MAPK/AP-1 and NF-κB in human gingival fibroblasts. Cytokine (2005) 29:159–68. doi: 10.1016/j.cyto.2004.10.009 15652448

[91] Scheres N Laine ML Sipos PM Bosch-Tijhof CJ Crielaard W De Vries TJ . Periodontal ligament and gingival fibroblasts from periodontitis patients are more active in interaction with porphyromonas gingivalis. J Periodontal Res (2011) 46:407–16. doi: 10.1111/j.1600-0765.2011.01353.x 21332474

[92] Leonardi R Talic NF Loreto C . MMP-13 (collagenase 3) immunolocalisation during initial orthodontic tooth movement in rats. Acta Histochemica (2007) 109:215–20. doi: 10.1016/j.acthis.2007.01.002 17350083

[93] Pan W Wang Q Chen Q . The cytokine network involved in the host immune response to periodontitis. Int J Oral Sci (2019) 11:30. doi: 10.1038/s41368-019-0064-z 31685798 PMC6828663

[94] Kondaiah P Pant I Khan I . Molecular pathways regulated by areca nut in the etiopathogenesis of oral submucous fibrosis. Periodontol 2000 (2019) 80:213–24. doi: 10.1111/prd.12266 31090136

[95] Rehman A Ali S Lone MA Atif M Hassona Y Prime SS . Areca nut alkaloids induce irreparable DNA damage and senescence in fibroblasts and may create a favourable environment for tumour progression. J Oral Pathol Med (2016) 45:365–72. doi: 10.1111/jop.12370 26414019

[96] Pitiyage GN Lim KP Gemenitzidis E Teh MT Waseem A Prime SS . Increased secretion of tissue inhibitors of metalloproteinases 1 and 2 (TIMPs -1 and -2) in fibroblasts are early indicators of oral sub-mucous fibrosis and ageing. J Oral Pathol Med (2012) 41:454–62. doi: 10.1111/j.1600-0714.2012.01129.x 22385081

[97] Sharma M Hunter KD Fonseca FP Radhakrishnan R . Emerging role of cellular senescence in the pathogenesis of oral submucous fibrosis and its malignant transformation. Head Neck (2021) 43:3153–64. doi: 10.1002/hed.26805 34227702

[98] Chen X Huang J Zhong L Ding C . Quantitative assessment of the associations between interleukin-8 polymorphisms and periodontitis susceptibility. J Periodontology (2015) 86:292–300. doi: 10.1902/jop.2014.140450 25299389

[99] Naruishi K Nishimura F Yamada-Naruishi H Omori K Yamaguchi M Takashiba S . C-jun n-terminal kinase (JNK) inhibitor, SP600125, blocks interleukin (IL)–6-induced vascular endothelial growth factor (VEGF) production: cyclosporine a partially mimics this inhibitory effect. Transplantation (2003) 76 (5):1380–2. doi: 10.1097/01.TP.0000085661.52980.95 14627919

[100] Sato K Suematsu A Okamoto K Yamaguchi A Morishita Y Kadono Y . Th17 functions as an osteoclastogenic helper T cell subset that links T cell activation and bone destruction. J Exp Med (2006) 203:2673–82. doi: 10.1084/jem.20061775 PMC2118166 17088434

[101] Takayanagi H Ogasawara K Hida S Chiba T Murata S Sato K . T-Cell-mediated regulation of osteoclastogenesis by signalling cross-talk between RANKL and IFN-γ. Nature (2000) 408:600–5. doi: 10.1038/35046102 11117749

[102] Azuma Y Kaji K Katogi R Takeshita S Kudo A . Tumor necrosis factor-α induces differentiation of and bone resorption by osteoclasts*. J Biol Chem (2000) 275:4858–64. doi: 10.1074/jbc.275.7.4858 10671521

[103] Zhou S Greenberger JS Epperly MW Goff JP Adler C Leboff MS . Age-related intrinsic changes in human bone-marrow-derived mesenchymal stem cells and their differentiation to osteoblasts. Aging Cell (2008) 7:335–43. doi: 10.1111/j.1474-9726.2008.00377.x PMC2398731 18248663

[104] Hwang YH Kim T Kim R Ha H . The natural product 6-gingerol inhibits inflammation-associated osteoclast differentiation *via* reduction of prostaglandin E₂ levels. Int J Mol Sci (2018) 19 (7):2068. doi: 10.3390/ijms19072068 PMC6073224 30013004

[105] Litwinoff E Hurtado Del Pozo C Ramasamy R Schmidt AM . Emerging targets for therapeutic development in diabetes and its complications: The RAGE signaling pathway. Clin Pharmacol Ther (2015) 98:135–44. doi: 10.1002/cpt.148 PMC4621004 25974754

[106] Mohan S Baylink D . Serum insulin-like growth factor binding protein (IGFBP)-4 and IGFBP-5 levels in aging and age-associated diseases. Endocrine (1997) 7:87–91. doi: 10.1007/BF02778070 9449039

[107] Hassona Y Cirillo N Lim KP Herman A Mellone M Thomas GJ . Progression of genotype-specific oral cancer leads to senescence of cancer-associated fibroblasts and is mediated by oxidative stress and TGF-β. Carcinogenesis (2013) 34:1286–95. doi: 10.1093/carcin/bgt035 23358854

[108] Chaffer CL Weinberg RA . A perspective on cancer cell metastasis. Science (2011) 331:1559–64. doi: 10.1126/science.1203543 21436443

[109] Balkwill F Charles KA Mantovani A . Smoldering and polarized inflammation in the initiation and promotion of malignant disease. Cancer Cell (2005) 7:211–7. doi: 10.1016/j.ccr.2005.02.013 15766659

[110] Bonnans C Chou J Werb Z . Remodelling the extracellular matrix in development and disease. Nat Rev Mol Cell Biol (2014) 15:786–801. doi: 10.1038/nrm3904 25415508 PMC4316204

[111] Park DG Woo BH Lee BJ Yoon S Cho Y Kim YD . Serum levels of interleukin-6 and titers of antibodies against porphyromonas gingivalis could be potential biomarkers for the diagnosis of oral squamous cell carcinoma. Int J Mol Sci (2019) 20 (11):2749. doi: 10.3390/ijms20112749 PMC6600294 31167516

[112] Qu Y He Y Yang Y Li S An W Li Z . ALDH3A1 acts as a prognostic biomarker and inhibits the epithelial mesenchymal transition of oral squamous cell carcinoma through IL-6/STAT3 signaling pathway. J Cancer (2020) 11:2621–31. doi: 10.7150/jca.40171 PMC7066020 32201532

[113] Liang S Hosur KB Domon H Hajishengallis G . Periodontal inflammation and bone loss in aged mice. J Periodontal Res (2010) 45:574–8. doi: 10.1111/j.1600-0765.2009.01245.x PMC2894296 20337897

[114] Miller CS Ding X Dawson Iii DR Ebersole JL . Salivary biomarkers for discriminating periodontitis in the presence of diabetes. J Clin Periodontology (2021) 48:216–25. doi: 10.1111/jcpe.13393 33098098

[115] Wang Q Zhou X Zhang P Zhao P Nie L Ji N . 25-hydroxyvitamin D(3) positively regulates periodontal inflammaging *via* SOCS3/STAT signaling in diabetic mice. Steroids (2020) 156:108570. doi: 10.1016/j.steroids.2019.108570 31917967

[116] Qin ZY Gu X Chen YL Liu JB Hou CX Lin SY . Toll−like receptor 4 activates the NLRP3 inflammasome pathway and periodontal inflammaging by inhibiting Bmi−1 expression. Int J Mol Med (2021) 47:137–50. doi: 10.3892/ijmm.2020.4787 PMC7723510 33236134

[117] Kato CN Barra SG Tavares NP Amaral TM Brasileiro CB Mesquita RA . Use of fractal analysis in dental images: a systematic review. Dentomaxillofac Radiol (2020) 49:20180457. doi: 10.1259/dmfr.20180457 31429597 PMC7026934

[118] Damanaki A Memmert S Nokhbehsaim M Sanyal A Gnad T Pfeifer A . Impact of obesity and aging on crestal alveolar bone height in mice. Ann Anat (2018) 218:227–35. doi: 10.1016/j.aanat.2018.04.005 29730468

[119] Aquino-Martinez R Eckhardt BA Rowsey JL Fraser DG Khosla S Farr JN . Senescent cells exacerbate chronic inflammation and contribute to periodontal disease progression in old mice. J Periodontol (2020) 92 (10):1483–95. doi: 10.1002/JPER.20-0529 PMC8281492 33341947

[120] Holguin N Brodt MD Silva MJ . Activation of wnt signaling by mechanical loading is impaired in the bone of old mice. J Bone Miner Res (2016) 31:2215–26. doi: 10.1002/jbmr.2900 PMC5397287 27357062

[121] Kumar M Nanavati R Modi TG Dobariya C . Oral cancer: Etiology and risk factors: A review. J Cancer Res Ther (2016) 12:458–63. doi: 10.4103/0973-1482.186696 27461593

[122] Feng Y Li Q Chen J Yi P Xu X Fan Y . Salivary protease spectrum biomarkers of oral cancer. Int J Oral Sci (2019) 11:7. doi: 10.1038/s41368-018-0032-z 30602733 PMC6315043

[123] Hsin CH Chou YE Yang SF Su SC Chuang YT Lin SH . MMP-11 promoted the oral cancer migration and Fak/Src activation. Oncotarget (2017) 8:32783–93. doi: 10.18632/oncotarget.15824 PMC5464827 28427180

[124] Ahmed Haji Omar A Haglund C Virolainen S Häyry V Atula T Kontio R . MMP-7, MMP-8, and MMP-9 in oral and cutaneous squamous cell carcinomas. Oral Surg Oral Med Oral Pathol Oral Radiol (2015) 119:459–67. doi: 10.1016/j.oooo.2014.12.019 25697929

